# μ-4,4′-Bipyridine-κ^2^ *N*:*N*′-bis­{[2-(3,5-dibromo-2-oxidobenzyl­ideneamino)-3-hydroxy­propanoato-κ^3^ *O*,*N*,*O*′]copper(II)} monohydrate

**DOI:** 10.1107/S1600536808018357

**Published:** 2008-06-21

**Authors:** Yong Liao Wang, Zheng Liu, Yuan Wang

**Affiliations:** a Key Laboratory of Non-ferrous Metal Materials and Processing Technology, Department of Materials and Chemical Engineering, Guilin University of Technology, Ministry of Education, Guilin 541004, People’s Republic of China

## Abstract

The title compound, [Cu_2_(C_10_H_7_Br_2_NO_4_)_2_(C_10_H_8_N_2_)]·H_2_O, is a binuclear copper(II) complex. Both Cu atoms are four-coordinate in a square-planar geometry. In addition, there is one water mol­ecule in the asymmetric unit. The crystal structure is stabilized by O—H⋯O and O—H⋯Br hydrogen bonds.

## Related literature

For related literature, see: Gao *et al.* (2005 ▶); Liang *et al.* (2006 ▶); Zhang *et al.* (2003 ▶).
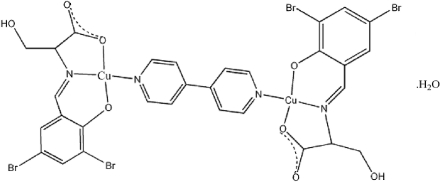


## Experimental

### 

#### Crystal data

[Cu_2_(C_10_H_7_Br_2_NO_4_)_2_(C_10_H_8_N_2_)]·H_2_O
*M* *_r_* = 1031.25
Monoclinic, 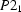

*a* = 7.3905 (7) Å
*b* = 11.3374 (16) Å
*c* = 19.943 (2) Å
β = 93.686 (2)°
*V* = 1667.6 (3) Å^3^
*Z* = 2
Mo *K*α radiation
μ = 6.13 mm^−1^
*T* = 298 (2) K
0.18 × 0.17 × 0.16 mm

#### Data collection

Bruker SMART CCD area-detector diffractometer
Absorption correction: multi-scan (*SADABS*; Sheldrick, 1996 ▶) *T*_min_ = 0.405, *T*_max_ = 0.441 (expected range = 0.345–0.375)
8428 measured reflections
5732 independent reflections
3029 reflections with *I* > 2σ(*I*)
*R*_int_ = 0.084

#### Refinement

*R*[*F* ^2^ > 2σ(*F* ^2^)] = 0.073
*wR*(*F* ^2^) = 0.187
*S* = 1.02
5732 reflections
442 parameters
1 restraint
H-atom parameters constrained
Δρ_max_ = 0.80 e Å^−3^
Δρ_min_ = −0.73 e Å^−3^
Absolute structure: Flack (1983 ▶), 5732 Friedel pairs
Flack parameter: 0.00 (3)

### 

Data collection: *SMART* (Bruker, 2004 ▶); cell refinement: *SAINT* (Bruker, 2004 ▶); data reduction: *SAINT*; program(s) used to solve structure: *SHELXS97* (Sheldrick, 2008 ▶); program(s) used to refine structure: *SHELXL97* (Sheldrick, 2008 ▶); molecular graphics: *SHELXTL* (Sheldrick, 2008 ▶); software used to prepare material for publication: *SHELXTL*.

## Supplementary Material

Crystal structure: contains datablocks global, I. DOI: 10.1107/S1600536808018357/bt2726sup1.cif

Structure factors: contains datablocks I. DOI: 10.1107/S1600536808018357/bt2726Isup2.hkl

Additional supplementary materials:  crystallographic information; 3D view; checkCIF report

## Figures and Tables

**Table 1:** Hydrogen-bond geometry (Å, °)

*D*—H⋯*A*	*D*—H	H⋯*A*	*D*⋯*A*	*D*—H⋯*A*
O3—H3⋯O2^i^	0.82	2.10	2.90 (2)	164
O7—H7⋯O6^ii^	0.82	1.93	2.73 (2)	165
O9—H9*A*⋯Br3^i^	0.85	2.57	3.415 (14)	171
O9—H9*B*⋯Br4^iii^	0.85	2.76	3.599 (15)	171

Symmetry codes: (i) 
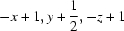
; (ii) 
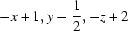
; (iii) 
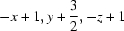
.

**Fig. 1.**
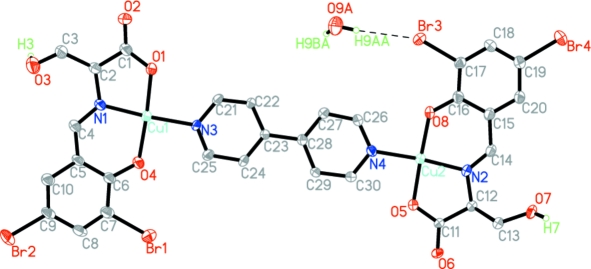
A view of (I), showing 30% probability displacement ellipsoids.

**Fig. 2.**
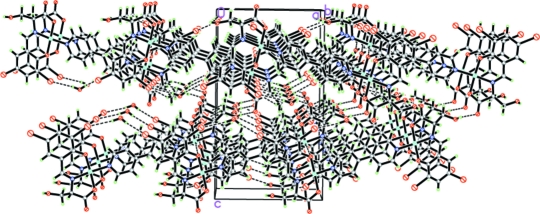
The three-dimensional network of (I) through hydrogen bonds.

## supplementary crystallographic information

### Comment

Amino acids are the basic unit of   proteins and enzymes, and they can form a series of amino acid Schiff-bases. Even though considerable work has been done on the complexes of amino acid Schiff-bases (Zhang *et al.*, 2003; Liang *et al.*, 2006; Gao *et al.*, 2005), there is not much reported on amino acid Schiff-base complexes containing 4,4'-bipyridine as an additional  ligand.

The coordination environment of Cu1 and Cu2 is almost equal, so we choose Cu1 for discussion. The distance of Cu1—N1 is 1.932 (16)Å, and the distance   Cu1—N3 is 2.038 (14)Å. The bond angle of four atoms coordinated with   Cu1 ion are as follows: O4—Cu1—N1 = 93.2 (6)°, N1—Cu1—O1 = 83.7 (6)°, O1—Cu1—N3 = 92.7 (6)°, N3—Cu1—O4 = 90.5 (6)°. The mean deviation from plane of the four atoms   coordinated to Cu1 is 0.0507 Å, showing that these four atoms lie in a common plane. The packing didagram  (Fig.2) shows that the   water molecules is connected to the complex through H–O···Br hydrogen bonds.  In addition, there are O–H···O hydrogen bonds stabilizing the crystal structure.

### Experimental

An ethanol solution (5 ml) containing 3,5-dibromo-2-hydroxy-benzaldehyde (0.140 g, 0.5 mmol) was added to an aqueous solution (5 ml) containing 2-amino-3-hydroxy-propionic acid (0.056 g, 0.5 mmol) and sodium hydroxide (0.040 g, 1 mmol). After stirring for 1 h, an aqueous solution of copper nitrate (0.120 g, 0.5 mmol) was added to the resulting solution and stirred for 2 h. At last, the ethanol solution (5 ml) containing 4, 4'-bipyridine (0.05 g, 0.025 mmol) was added. The green solution was filtrated. After five days, green block shaped crystals were obtained by slow evaporation of the filtrate (yield: 36.5%, based on Cu).

### Refinement

Water H atoms were located in a difference Fourier map and were allowed to ride on the O atom, with *U*_iso_(H) = 1.5*U*_eq_(O). All other H atomswere positioned geometrically and refined as riding, with C–H = 0.93 Å and with *U*_iso_(H) = 1.2 *U*_eq_(C).

### Crystal data

**Table:**

[Cu_2_(C_10_H_7_Br_2_N_1_O_4_)_2_(C_10_H_8_N_2_)]·H_2_O	*F*_000_ = 1004
*M**_r_* = 1031.25	*D*_x_ = 2.054 Mg m^−^^3^
Monoclinic, *P*2_1_	Mo *K*α radiation λ = 0.71073 Å
Hall symbol: P 2yb	Cell parameters from 1685 reflections
*a* = 7.3905 (7) Å	θ = 2.7–24.9º
*b* = 11.3374 (16) Å	µ = 6.13 mm^−^^1^
*c* = 19.943 (2) Å	*T* = 298 (2) K
β = 93.686 (2)º	Block, green
*V* = 1667.6 (3) Å^3^	0.18 × 0.17 × 0.16 mm
*Z* = 2	

### Data collection

**Table:**

Bruker SMART CCD area-detector diffractometer	5732 independent reflections
Radiation source: fine-focus sealed tube	3029 reflections with *I* > 2σ(*I*)
Monochromator: graphite	*R*_int_ = 0.084
*T* = 298(2) K	θ_max_ = 25.0º
φ and ω scans	θ_min_ = 2.1º
Absorption correction: multi-scan(SADABS; Sheldrick, 1996)	*h* = −5→8
*T*_min_ = 0.405, *T*_max_ = 0.441	*k* = −13→13
8428 measured reflections	*l* = −23→23

### Refinement

**Table:**

Refinement on *F*^2^	Hydrogen site location: inferred from neighbouring sites
Least-squares matrix: full	H-atom parameters constrained
*R*[*F*^2^ > 2σ(*F*^2^)] = 0.073	*w* = 1/[σ^2^(*F*_o_^2^) + (0.0693*P*)^2^] where *P* = (*F*_o_^2^ + 2*F*_c_^2^)/3
*wR*(*F*^2^) = 0.187	(Δ/σ)_max_ = 0.001
*S* = 1.02	Δρ_max_ = 0.80 e Å^−^^3^
5732 reflections	Δρ_min_ = −0.73 e Å^−^^3^
442 parameters	Extinction correction: none
1 restraint	Absolute structure: Flack (1983), 2625 Friedel pairs
Primary atom site location: structure-invariant direct methods	Flack parameter: 0.00 (3)
Secondary atom site location: difference Fourier map	

### Special details

**Table:**

Geometry. All e.s.d.'s (except the e.s.d. in the dihedral angle between two l.s. planes) are estimated using the full covariance matrix. The cell e.s.d.'s are taken into account individually in the estimation of e.s.d.'s in distances, angles and torsion angles; correlations between e.s.d.'s in cell parameters are only used when they are defined by crystal symmetry. An approximate (isotropic) treatment of cell e.s.d.'s is used for estimating e.s.d.'s involving l.s. planes.
Refinement. Refinement of *F*^2^ against ALL reflections. The weighted *R*-factor *wR* and goodness of fit *S* are based on *F*^2^, conventional *R*-factors *R* are based on *F*, with *F* set to zero for negative *F*^2^. The threshold expression of *F*^2^ > σ(*F*^2^) is used only for calculating *R*-factors(gt) *etc*. and is not relevant to the choice of reflections for refinement. *R*-factors based on *F*^2^ are statistically about twice as large as those based on *F*, and *R*- factors based on ALL data will be even larger.

### Fractional atomic coordinates and isotropic or equivalent isotropic displacement parameters (Å^2^)

**Table:**

	*x*	*y*	*z*	*U*_iso_*/*U*_eq_	
Cu1	0.6425 (3)	0.82294 (19)	0.68048 (12)	0.0410 (6)	
Cu2	0.3052 (3)	−0.0991 (2)	0.82661 (12)	0.0400 (6)	
Br1	0.8208 (3)	0.8393 (2)	0.91853 (11)	0.0580 (6)	
Br2	1.0514 (3)	1.3118 (2)	0.88646 (14)	0.0752 (8)	
Br3	0.1238 (3)	−0.11439 (18)	0.58941 (10)	0.0531 (6)	
Br4	−0.0792 (3)	−0.5930 (2)	0.61848 (12)	0.0656 (7)	
N1	0.678 (2)	0.9816 (14)	0.6484 (8)	0.042 (4)	
N2	0.251 (2)	−0.2541 (14)	0.8606 (8)	0.042 (4)	
N3	0.589 (2)	0.6542 (12)	0.7086 (8)	0.037 (4)	
N4	0.3702 (19)	0.0648 (13)	0.7989 (8)	0.036 (4)	
O1	0.5913 (18)	0.7883 (11)	0.5864 (7)	0.047 (4)	
O2	0.558 (2)	0.8659 (13)	0.4829 (7)	0.067 (5)	
O3	0.724 (2)	1.1969 (14)	0.5553 (8)	0.081 (5)	
H3	0.6332	1.2329	0.5414	0.122*	
O4	0.7108 (17)	0.8640 (11)	0.7719 (6)	0.049 (4)	
O5	0.3548 (17)	−0.0627 (10)	0.9202 (6)	0.043 (4)	
O6	0.3950 (19)	−0.1389 (12)	1.0224 (6)	0.055 (4)	
O7	0.3448 (19)	−0.4755 (12)	0.9412 (8)	0.068 (4)	
H7	0.4236	−0.5264	0.9447	0.102*	
O8	0.2331 (17)	−0.1390 (10)	0.7358 (7)	0.046 (4)	
O9	0.820 (2)	0.6748 (12)	0.4525 (8)	0.095 (5)	
H9A	0.8438	0.6055	0.4391	0.114*	
H9B	0.8877	0.7233	0.4332	0.114*	
C1	0.578 (3)	0.8763 (19)	0.5442 (11)	0.053 (5)	
C2	0.612 (3)	0.9974 (17)	0.5764 (10)	0.048 (5)	
H2	0.4911	1.0326	0.5780	0.058*	
C3	0.714 (3)	1.0800 (16)	0.5322 (10)	0.057 (6)	
H3A	0.8362	1.0501	0.5292	0.068*	
H3B	0.6553	1.0794	0.4873	0.068*	
C4	0.759 (3)	1.0674 (17)	0.6838 (10)	0.047 (5)	
H4	0.7762	1.1387	0.6619	0.056*	
C5	0.821 (3)	1.0595 (17)	0.7508 (10)	0.044 (5)	
C6	0.793 (3)	0.9601 (18)	0.7942 (11)	0.043 (5)	
C7	0.853 (3)	0.9737 (18)	0.8614 (11)	0.050 (6)	
C8	0.928 (3)	1.0682 (19)	0.8908 (11)	0.049 (6)	
H8	0.9611	1.0699	0.9365	0.059*	
C9	0.954 (3)	1.1678 (18)	0.8481 (11)	0.048 (6)	
C10	0.900 (3)	1.1637 (19)	0.7819 (11)	0.049 (5)	
H10	0.9140	1.2305	0.7557	0.058*	
C11	0.351 (2)	−0.1498 (17)	0.9624 (10)	0.043 (5)	
C12	0.282 (3)	−0.2650 (16)	0.9346 (10)	0.044 (5)	
H12	0.1672	−0.2841	0.9540	0.053*	
C13	0.424 (3)	−0.3636 (15)	0.9540 (10)	0.050 (5)	
H13A	0.5294	−0.3543	0.9280	0.060*	
H13B	0.4629	−0.3570	1.0013	0.060*	
C14	0.171 (2)	−0.3387 (17)	0.8278 (10)	0.040 (5)	
H14	0.1344	−0.4035	0.8521	0.048*	
C15	0.132 (3)	−0.3404 (17)	0.7536 (10)	0.041 (5)	
C16	0.155 (3)	−0.2372 (17)	0.7129 (11)	0.041 (5)	
C17	0.100 (2)	−0.2486 (17)	0.6457 (10)	0.039 (5)	
C18	0.029 (2)	−0.3534 (17)	0.6184 (11)	0.046 (5)	
H18	−0.0080	−0.3568	0.5729	0.055*	
C19	0.013 (3)	−0.4503 (17)	0.6576 (11)	0.044 (5)	
C20	0.056 (2)	−0.4448 (17)	0.7274 (10)	0.043 (5)	
H20	0.0349	−0.5086	0.7551	0.051*	
C21	0.520 (3)	0.5759 (16)	0.6625 (10)	0.039 (5)	
H21	0.5016	0.6008	0.6181	0.047*	
C22	0.477 (2)	0.4650 (16)	0.6773 (10)	0.038 (5)	
H22	0.4247	0.4162	0.6439	0.046*	
C23	0.509 (2)	0.4221 (15)	0.7418 (9)	0.034 (4)	
C24	0.579 (2)	0.4999 (16)	0.7890 (10)	0.040 (5)	
H24	0.6018	0.4758	0.8333	0.048*	
C25	0.618 (3)	0.6195 (16)	0.7698 (10)	0.041 (5)	
H25	0.6642	0.6724	0.8022	0.049*	
C26	0.331 (3)	0.1090 (16)	0.7361 (10)	0.041 (5)	
H26	0.2729	0.0586	0.7048	0.050*	
C27	0.368 (2)	0.2229 (16)	0.7147 (10)	0.040 (5)	
H27	0.3377	0.2472	0.6709	0.048*	
C28	0.454 (2)	0.2985 (16)	0.7613 (9)	0.039 (5)	
C29	0.498 (2)	0.2568 (16)	0.8271 (10)	0.039 (5)	
H29	0.5570	0.3058	0.8589	0.047*	
C30	0.452 (2)	0.1379 (16)	0.8441 (10)	0.041 (5)	
H30	0.4801	0.1110	0.8876	0.050*	

### Atomic displacement parameters (Å^2^)

**Table:**

	*U*^11^	*U*^22^	*U*^33^	*U*^12^	*U*^13^	*U*^23^
Cu1	0.0588 (15)	0.0261 (13)	0.0369 (14)	−0.0032 (12)	−0.0069 (12)	0.0037 (12)
Cu2	0.0560 (14)	0.0274 (13)	0.0353 (14)	−0.0029 (12)	−0.0063 (11)	0.0035 (12)
Br1	0.0639 (13)	0.0598 (15)	0.0494 (14)	0.0004 (11)	−0.0033 (10)	0.0105 (12)
Br2	0.0904 (18)	0.0616 (18)	0.0719 (17)	−0.0270 (15)	−0.0074 (13)	−0.0250 (16)
Br3	0.0715 (14)	0.0456 (13)	0.0410 (13)	−0.0020 (11)	−0.0063 (10)	0.0104 (10)
Br4	0.0863 (16)	0.0439 (14)	0.0649 (16)	−0.0157 (13)	−0.0071 (12)	−0.0127 (14)
N1	0.054 (10)	0.032 (10)	0.038 (10)	−0.005 (8)	−0.008 (8)	−0.004 (8)
N2	0.058 (11)	0.027 (9)	0.039 (11)	0.003 (8)	−0.003 (8)	0.007 (8)
N3	0.059 (10)	0.020 (8)	0.030 (10)	0.000 (7)	−0.004 (8)	0.006 (8)
N4	0.047 (10)	0.029 (9)	0.032 (10)	−0.006 (7)	0.007 (8)	−0.005 (8)
O1	0.070 (10)	0.027 (8)	0.044 (9)	−0.004 (6)	−0.007 (7)	0.003 (7)
O2	0.110 (11)	0.052 (12)	0.037 (9)	−0.016 (8)	−0.009 (8)	0.000 (8)
O3	0.103 (12)	0.052 (10)	0.087 (13)	−0.017 (9)	−0.015 (10)	0.004 (10)
O4	0.070 (9)	0.030 (8)	0.044 (8)	−0.005 (6)	−0.012 (7)	−0.003 (6)
O5	0.065 (9)	0.027 (8)	0.037 (8)	−0.004 (6)	−0.007 (7)	0.001 (6)
O6	0.106 (11)	0.039 (10)	0.020 (8)	−0.005 (7)	−0.001 (7)	0.004 (6)
O7	0.097 (12)	0.032 (8)	0.069 (11)	−0.011 (7)	−0.033 (8)	0.018 (8)
O8	0.064 (9)	0.026 (8)	0.046 (8)	−0.009 (6)	−0.003 (7)	0.003 (6)
O9	0.136 (15)	0.049 (9)	0.099 (13)	−0.016 (9)	−0.004 (11)	−0.008 (8)
C1	0.072 (14)	0.037 (13)	0.047 (13)	−0.004 (10)	−0.009 (11)	−0.005 (12)
C2	0.067 (14)	0.039 (12)	0.038 (12)	−0.005 (10)	−0.010 (11)	−0.003 (10)
C3	0.073 (15)	0.043 (12)	0.052 (14)	−0.009 (11)	−0.015 (11)	0.006 (10)
C4	0.061 (14)	0.035 (12)	0.044 (13)	−0.005 (10)	−0.004 (11)	−0.008 (10)
C5	0.059 (14)	0.035 (12)	0.038 (13)	−0.003 (9)	−0.009 (10)	−0.013 (10)
C6	0.055 (13)	0.033 (12)	0.040 (13)	0.002 (9)	−0.011 (10)	−0.005 (10)
C7	0.060 (14)	0.041 (13)	0.046 (14)	0.006 (10)	−0.012 (11)	−0.008 (11)
C8	0.056 (14)	0.043 (13)	0.047 (14)	0.008 (10)	−0.018 (11)	−0.013 (11)
C9	0.057 (14)	0.038 (13)	0.048 (14)	0.006 (9)	−0.014 (11)	−0.017 (11)
C10	0.060 (14)	0.040 (12)	0.045 (14)	0.001 (10)	−0.004 (11)	−0.006 (11)
C11	0.063 (13)	0.030 (12)	0.035 (11)	−0.004 (9)	−0.016 (9)	0.005 (10)
C12	0.061 (13)	0.032 (11)	0.037 (12)	−0.004 (9)	−0.011 (10)	0.006 (9)
C13	0.076 (15)	0.031 (11)	0.041 (12)	0.000 (10)	−0.013 (11)	0.005 (9)
C14	0.047 (12)	0.031 (11)	0.043 (13)	0.000 (9)	−0.001 (10)	0.009 (10)
C15	0.045 (12)	0.034 (12)	0.042 (13)	0.002 (9)	0.004 (10)	0.004 (10)
C16	0.045 (12)	0.032 (12)	0.046 (14)	0.004 (9)	−0.001 (10)	−0.001 (10)
C17	0.039 (11)	0.035 (12)	0.041 (13)	0.004 (8)	−0.004 (9)	0.005 (10)
C18	0.053 (13)	0.042 (13)	0.042 (13)	−0.003 (10)	−0.001 (11)	−0.007 (10)
C19	0.048 (12)	0.036 (12)	0.047 (14)	0.001 (9)	0.003 (10)	−0.004 (11)
C20	0.049 (12)	0.031 (12)	0.047 (14)	0.004 (9)	−0.003 (10)	0.002 (10)
C21	0.057 (13)	0.031 (12)	0.029 (11)	0.002 (9)	0.002 (10)	0.006 (9)
C22	0.055 (12)	0.024 (11)	0.034 (13)	−0.003 (9)	−0.010 (9)	0.005 (9)
C23	0.050 (11)	0.023 (11)	0.028 (11)	0.000 (8)	−0.005 (9)	0.000 (9)
C24	0.057 (13)	0.028 (12)	0.035 (12)	−0.001 (9)	−0.003 (10)	0.009 (9)
C25	0.057 (13)	0.032 (11)	0.032 (12)	0.004 (9)	0.000 (10)	0.003 (9)
C26	0.056 (13)	0.031 (11)	0.037 (13)	−0.010 (9)	−0.001 (10)	−0.001 (10)
C27	0.056 (13)	0.032 (12)	0.032 (12)	0.001 (9)	−0.002 (10)	0.002 (9)
C28	0.053 (12)	0.027 (12)	0.035 (12)	0.001 (9)	−0.002 (10)	0.000 (10)
C29	0.055 (13)	0.028 (12)	0.033 (13)	−0.003 (9)	−0.012 (9)	−0.002 (9)
C30	0.057 (13)	0.036 (12)	0.031 (12)	−0.006 (9)	−0.004 (10)	0.001 (9)

### Geometric parameters (Å, °)

**Table:**

Cu1—O4	1.917 (13)	C5—C6	1.44 (3)
Cu1—O1	1.930 (13)	C6—C7	1.39 (3)
Cu1—N1	1.932 (16)	C7—C8	1.32 (3)
Cu1—N3	2.038 (14)	C8—C9	1.44 (3)
Cu2—O8	1.909 (13)	C8—H8	0.9300
Cu2—O5	1.924 (13)	C9—C10	1.36 (3)
Cu2—N2	1.934 (16)	C10—H10	0.9300
Cu2—N4	2.006 (15)	C11—C12	1.50 (3)
Br1—C7	1.93 (2)	C12—C13	1.56 (2)
Br2—C9	1.92 (2)	C12—H12	0.9800
Br3—C17	1.905 (19)	C13—H13A	0.9700
Br4—C19	1.90 (2)	C13—H13B	0.9700
N1—C4	1.32 (2)	C14—C15	1.49 (3)
N1—C2	1.50 (2)	C14—H14	0.9300
N2—C14	1.28 (2)	C15—C20	1.40 (3)
N2—C12	1.48 (2)	C15—C16	1.44 (3)
N3—C25	1.29 (2)	C16—C17	1.38 (3)
N3—C21	1.36 (2)	C17—C18	1.40 (3)
N4—C30	1.34 (2)	C18—C19	1.36 (3)
N4—C26	1.36 (2)	C18—H18	0.9300
O1—C1	1.30 (2)	C19—C20	1.41 (3)
O2—C1	1.23 (2)	C20—H20	0.9300
O3—C3	1.40 (2)	C21—C22	1.34 (2)
O3—H3	0.8200	C21—H21	0.9300
O4—C6	1.31 (2)	C22—C23	1.38 (2)
O5—C11	1.30 (2)	C22—H22	0.9300
O6—C11	1.23 (2)	C23—C24	1.37 (2)
O7—C13	1.41 (2)	C23—C28	1.517 (18)
O7—H7	0.8200	C24—C25	1.44 (2)
O8—C16	1.32 (2)	C24—H24	0.9300
O9—H9A	0.8500	C25—H25	0.9300
O9—H9B	0.8500	C26—C27	1.39 (2)
C1—C2	1.53 (3)	C26—H26	0.9300
C2—C3	1.52 (3)	C27—C28	1.39 (2)
C2—H2	0.9800	C27—H27	0.9300
C3—H3A	0.9700	C28—C29	1.41 (2)
C3—H3B	0.9700	C29—C30	1.44 (2)
C4—C5	1.39 (3)	C29—H29	0.9300
C4—H4	0.9300	C30—H30	0.9300
C5—C10	1.44 (3)		
			
O4—Cu1—O1	175.3 (6)	O6—C11—O5	122.7 (18)
O4—Cu1—N1	93.2 (6)	O6—C11—C12	120.8 (18)
O1—Cu1—N1	83.7 (6)	O5—C11—C12	116.4 (16)
O4—Cu1—N3	90.5 (6)	N2—C12—C11	108.8 (15)
O1—Cu1—N3	92.7 (6)	N2—C12—C13	111.3 (16)
N1—Cu1—N3	175.6 (6)	C11—C12—C13	109.1 (15)
O8—Cu2—O5	174.5 (6)	N2—C12—H12	109.2
O8—Cu2—N2	93.8 (6)	C11—C12—H12	109.2
O5—Cu2—N2	83.4 (6)	C13—C12—H12	109.2
O8—Cu2—N4	90.9 (6)	O7—C13—C12	109.5 (15)
O5—Cu2—N4	92.0 (6)	O7—C13—H13A	109.8
N2—Cu2—N4	175.3 (7)	C12—C13—H13A	109.8
C4—N1—C2	122.1 (17)	O7—C13—H13B	109.8
C4—N1—Cu1	125.1 (14)	C12—C13—H13B	109.8
C2—N1—Cu1	112.7 (12)	H13A—C13—H13B	108.2
C14—N2—C12	118.8 (16)	N2—C14—C15	124.7 (18)
C14—N2—Cu2	126.8 (14)	N2—C14—H14	117.7
C12—N2—Cu2	113.7 (12)	C15—C14—H14	117.7
C25—N3—C21	118.4 (16)	C20—C15—C16	122.5 (18)
C25—N3—Cu1	121.7 (13)	C20—C15—C14	115.4 (18)
C21—N3—Cu1	119.9 (12)	C16—C15—C14	121.8 (18)
C30—N4—C26	116.9 (16)	O8—C16—C17	120.6 (19)
C30—N4—Cu2	119.5 (12)	O8—C16—C15	123.6 (19)
C26—N4—Cu2	123.5 (12)	C17—C16—C15	115.6 (18)
C1—O1—Cu1	118.3 (12)	C16—C17—C18	122.6 (19)
C3—O3—H3	109.5	C16—C17—Br3	117.7 (15)
C6—O4—Cu1	127.8 (13)	C18—C17—Br3	119.7 (16)
C11—O5—Cu2	117.1 (11)	C19—C18—C17	120 (2)
C13—O7—H7	109.5	C19—C18—H18	119.8
C16—O8—Cu2	128.1 (13)	C17—C18—H18	119.8
H9A—O9—H9B	108.6	C18—C19—C20	120.7 (19)
O2—C1—O1	124.7 (19)	C18—C19—Br4	119.5 (16)
O2—C1—C2	121 (2)	C20—C19—Br4	119.7 (15)
O1—C1—C2	114.4 (17)	C15—C20—C19	117.8 (19)
N1—C2—C3	119.0 (17)	C15—C20—H20	121.1
N1—C2—C1	109.2 (17)	C19—C20—H20	121.1
C3—C2—C1	112.6 (17)	C22—C21—N3	123.6 (18)
N1—C2—H2	104.9	C22—C21—H21	118.2
C3—C2—H2	104.9	N3—C21—H21	118.2
C1—C2—H2	104.9	C21—C22—C23	120.5 (19)
O3—C3—C2	114.2 (17)	C21—C22—H22	119.7
O3—C3—H3A	108.7	C23—C22—H22	119.7
C2—C3—H3A	108.7	C24—C23—C22	116.7 (17)
O3—C3—H3B	108.7	C24—C23—C28	121.0 (14)
C2—C3—H3B	108.7	C22—C23—C28	122.1 (14)
H3A—C3—H3B	107.6	C23—C24—C25	119.8 (18)
N1—C4—C5	125.1 (19)	C23—C24—H24	120.1
N1—C4—H4	117.5	C25—C24—H24	120.1
C5—C4—H4	117.5	N3—C25—C24	121.1 (18)
C4—C5—C10	117.4 (19)	N3—C25—H25	119.5
C4—C5—C6	125.4 (19)	C24—C25—H25	119.5
C10—C5—C6	116.7 (18)	N4—C26—C27	125.9 (18)
O4—C6—C7	122 (2)	N4—C26—H26	117.1
O4—C6—C5	121.6 (18)	C27—C26—H26	117.1
C7—C6—C5	116.3 (18)	C28—C27—C26	117.3 (19)
C8—C7—C6	128 (2)	C28—C27—H27	121.4
C8—C7—Br1	116.2 (16)	C26—C27—H27	121.4
C6—C7—Br1	116.0 (15)	C27—C28—C29	119.0 (18)
C7—C8—C9	116 (2)	C27—C28—C23	121.1 (14)
C7—C8—H8	121.9	C29—C28—C23	119.8 (14)
C9—C8—H8	121.9	C28—C29—C30	119.3 (18)
C10—C9—C8	121 (2)	C28—C29—H29	120.4
C10—C9—Br2	119.8 (17)	C30—C29—H29	120.4
C8—C9—Br2	119.6 (15)	N4—C30—C29	121.6 (18)
C9—C10—C5	122 (2)	N4—C30—H30	119.2
C9—C10—H10	118.9	C29—C30—H30	119.2
C5—C10—H10	118.9		
			
O4—Cu1—N1—C4	11.7 (16)	Cu2—O5—C11—C12	−9(2)
O1—Cu1—N1—C4	−164.7 (16)	C14—N2—C12—C11	−171.5 (16)
O4—Cu1—N1—C2	−171.5 (13)	Cu2—N2—C12—C11	−1(2)
O1—Cu1—N1—C2	12.0 (13)	C14—N2—C12—C13	68 (2)
O8—Cu2—N2—C14	−8.0 (17)	Cu2—N2—C12—C13	−121.0 (14)
O5—Cu2—N2—C14	167.2 (17)	O6—C11—C12—N2	−176.3 (17)
O8—Cu2—N2—C12	−177.9 (13)	O5—C11—C12—N2	6(3)
O5—Cu2—N2—C12	−2.7 (12)	O6—C11—C12—C13	−55 (3)
O4—Cu1—N3—C25	−3.0 (15)	O5—C11—C12—C13	127.6 (17)
O1—Cu1—N3—C25	173.6 (14)	N2—C12—C13—O7	−73 (2)
O4—Cu1—N3—C21	176.1 (14)	C11—C12—C13—O7	167.3 (17)
O1—Cu1—N3—C21	−7.3 (14)	C12—N2—C14—C15	−179.4 (17)
O8—Cu2—N4—C30	−173.2 (13)	Cu2—N2—C14—C15	11 (3)
O5—Cu2—N4—C30	11.5 (14)	N2—C14—C15—C20	175.3 (17)
O8—Cu2—N4—C26	8.9 (15)	N2—C14—C15—C16	−11 (3)
O5—Cu2—N4—C26	−166.4 (15)	Cu2—O8—C16—C17	175.6 (13)
N1—Cu1—O1—C1	−8.0 (14)	Cu2—O8—C16—C15	−9(3)
N3—Cu1—O1—C1	169.3 (14)	C20—C15—C16—O8	−177.2 (17)
N1—Cu1—O4—C6	−14.4 (16)	C14—C15—C16—O8	9(3)
N3—Cu1—O4—C6	168.0 (15)	C20—C15—C16—C17	−1(3)
N2—Cu2—O5—C11	6.4 (13)	C14—C15—C16—C17	−174.9 (17)
N4—Cu2—O5—C11	−172.7 (13)	O8—C16—C17—C18	175.4 (17)
N2—Cu2—O8—C16	6.8 (15)	C15—C16—C17—C18	−1(3)
N4—Cu2—O8—C16	−173.8 (15)	O8—C16—C17—Br3	−5(2)
Cu1—O1—C1—O2	174.8 (16)	C15—C16—C17—Br3	179.1 (13)
Cu1—O1—C1—C2	2(2)	C16—C17—C18—C19	−1(3)
C4—N1—C2—C3	32 (3)	Br3—C17—C18—C19	179.2 (14)
Cu1—N1—C2—C3	−145.0 (15)	C17—C18—C19—C20	5(3)
C4—N1—C2—C1	163.1 (17)	C17—C18—C19—Br4	−178.5 (14)
Cu1—N1—C2—C1	−14 (2)	C16—C15—C20—C19	5(3)
O2—C1—C2—N1	−165.5 (18)	C14—C15—C20—C19	178.6 (16)
O1—C1—C2—N1	8(2)	C18—C19—C20—C15	−6(3)
O2—C1—C2—C3	−31 (3)	Br4—C19—C20—C15	176.9 (14)
O1—C1—C2—C3	142.5 (19)	C25—N3—C21—C22	1(3)
N1—C2—C3—O3	−59 (3)	Cu1—N3—C21—C22	−178.0 (15)
C1—C2—C3—O3	171.1 (17)	N3—C21—C22—C23	−3(3)
C2—N1—C4—C5	178.9 (19)	C21—C22—C23—C24	2(3)
Cu1—N1—C4—C5	−5(3)	C21—C22—C23—C28	177.2 (16)
N1—C4—C5—C10	−177.6 (18)	C22—C23—C24—C25	−1(3)
N1—C4—C5—C6	−5(3)	C28—C23—C24—C25	−175.5 (15)
Cu1—O4—C6—C7	−172.9 (14)	C21—N3—C25—C24	1(3)
Cu1—O4—C6—C5	10 (3)	Cu1—N3—C25—C24	179.8 (13)
C4—C5—C6—O4	3(3)	C23—C24—C25—N3	−1(3)
C10—C5—C6—O4	175.2 (17)	C30—N4—C26—C27	0(3)
C4—C5—C6—C7	−174.8 (19)	Cu2—N4—C26—C27	178.3 (14)
C10—C5—C6—C7	−2(3)	N4—C26—C27—C28	0(3)
C5—C6—C7—C8	2(3)	C26—C27—C28—C29	0(3)
O4—C6—C7—Br1	4(3)	C26—C27—C28—C23	177.0 (15)
C5—C6—C7—Br1	−178.7 (14)	C24—C23—C28—C27	174.5 (19)
C6—C7—C8—C9	−1(3)	C22—C23—C28—C27	0(2)
Br1—C7—C8—C9	179.1 (14)	C24—C23—C28—C29	−9(2)
C7—C8—C9—C10	2(3)	C22—C23—C28—C29	176.6 (19)
C7—C8—C9—Br2	177.2 (15)	C27—C28—C29—C30	−1(3)
C8—C9—C10—C5	−3(3)	C23—C28—C29—C30	−177.4 (16)
Br2—C9—C10—C5	−178.0 (15)	C26—N4—C30—C29	−1(3)
C4—C5—C10—C9	175.9 (19)	Cu2—N4—C30—C29	−178.8 (14)
C6—C5—C10—C9	3(3)	C28—C29—C30—N4	1(3)
Cu2—O5—C11—O6	173.7 (14)		

### Hydrogen-bond geometry (Å, °)

**Table:**

*D*—H···*A*	*D*—H	H···*A*	*D*···*A*	*D*—H···*A*
O3—H3···O2^i^	0.82	2.10	2.90 (2)	164
O7—H7···O6^ii^	0.82	1.93	2.73 (2)	165
O9—H9A···Br3^i^	0.85	2.57	3.415 (14)	171
O9—H9B···Br4^iii^	0.85	2.76	3.599 (15)	171

Symmetry codes: (i) −*x*+1, *y*+1/2, −*z*+1; (ii) −*x*+1, *y*−1/2, −*z*+2; (iii) −*x*+1, *y*+3/2, −*z*+1.
